# The Use and Abuse of Arsenious Acids as an Agent for Destroying the Dental Pulp and Nerve, Curing the Tooth-Ache

**Published:** 1851-07

**Authors:** A. C. Castle

**Affiliations:** Surgeon Dentist, New York.


### ARTICLE XXVIII.

*The Use and Abuse of Arsenious Acids as an Agent for Destroying the Dental Pulp and Nerve, Curing the Toothache.* By A. C. Castle, M. D., Surgeon Dentist, New York.

The author points out the terrible results of the indiscriminate use of large quantities of arsenious acid for the destruction of dental pulp. He shows, that however carefully and completely it may be preserved within the decayed cavity of a tooth from direct communication with the process of insalivation, it will gradually destroy the nervous fibrils within the fangs of the tooth, and thence penetrating to the periosteum and alveolus, will produce its characteristic and fearful effects on these structures. Instead of relieving toothache, the first consequence of its application is the increase of pain to a frightful and almost unbearable extent. Such torture cannot, however, remain long, without producing intense inflammation, which in most instances is followed by exfoliation of the alveolus, and its concomitant miseries. Dr. Castle, however, himself approves of the use of arsenious acids in very minute quantities, but not until after the nervous pulp has been paralyzed and destroyed by an agent more sudden and violent in its action. His plan is first to remove the diseased dentine from contact with the nerve; then to force a small wire of gold, which has been dipped in nitric acid, as far down as possible into the cavity of the tooth, which is attended with very little pain; he next inserts a pill of white soap, containing one-fiftieth of a grain of arsenious acid, and covers this with wax or cotton dipped in creosote. After a week or two, the tooth, no longer tender, is fit to receive the stuffing of amalgam or gold leaf. The author proceeds to detail three cases, in which arsenious acid was freely applied: extreme pain was occasioned, followed, in the two latter cases, by violent inflammation and exfoliation of the alveolus; and he states that in fifty per cent. of the cases so treated, abscesses formed at the apices of the fangs; and that in another thirty per cent., the teeth were eventually obliged to be removed, from their acting as foreign bodies, and causing much irritation. The author then remarks—

"I have never found that the action of arsenic upon the dental tissues was in any way modified, in whatsoever mixture or combination it may have been exhibited, whether intended as 'emollient,' 'palliative,' or 'preventive,' any more than these combinations could or would be expected to produce such results were they to be taken internally in cases of suicide, or administered by the murderer as a cover, or to prevent the usual symptoms of arsenic in such cases."

We are inclined to believe that, entertaining these opinions, Dr. Castle is advising a mode of treatment which a matured judgment should consider as too dangerous, under any circumstances, for general use.

The author has never found it necessary to repeat the application of the arsenious acid; but with every care and attention, we are inclined to agree very cordially in the concluding paragraph of the paper:—

"A practice at all times to be reprehended, as being alike disastrous to the tooth as well as the health and comfort of the patient."—*London Lancet.*

